# Azaperone and xylazine: A pharmacological combination to facilitate captive deer management for red brocket deer (*Mazama americana*)

**DOI:** 10.1371/journal.pone.0220288

**Published:** 2019-08-02

**Authors:** Adriano B. Carregaro, Bárbara G. Ferrari, André N. E. da Silva, Nathalia V. Xavier, José M. B. Duarte

**Affiliations:** 1 Department of Veterinary Medicine, Faculty of Animal Science and Food Engineering, University of Sao Paulo, Pirassununga, São Paulo, Brazil; 2 Department of Animal Science—Deer Research and Conservation Center (NUPECCE), Faculty of Agrarian and Veterinary Sciences, São Paulo State University (UNESP), Jaboticabal, São Paulo, Brazil; University of Bari, ITALY

## Abstract

The care and management of deer in captivity is challenging, especially in the case of red brocket deer (*Mazama americana*), whose routine management using physical restraint is difficult. Our study evaluated the effects of azaperone and xylazine combination for immobilizing red brocket deer and allow for the standard capture and handling protocols (e.g., biological material, horn cutting, and trimming) to be conducted safely. Six adult, captive, red brocket deer received an intramuscular injection of either 1 mg/kg azaperone and 0.5 mg/kg xylazine (AX0.5) or 1 mg/kg azaperone and 1 mg/kg xylazine (AX1.0). Sedation latency, sternal recumbency, safe handling, and quality of the sedation were evaluated to provide an overview of how the immobilizing drugs affected managing the species in captivity. Additionally, heart rate, respiratory rate, mean arterial pressure, rectal temperature, pH, PaO_2_, PaCO_2_, SaO_2_, HCO_3_^-^, BE, Na^+^, K^+^ and serum lactate were also measured. The latency period of the animals in the AX0.5 group was greater than that of the animals in the AX1.0 group (7 ± 6.6 min vs. 5 ± 2.0 min), as was the time for them to assume sternal recumbency (12 ± 9.7 min vs. 6 ± 3.1 min). However, the time after the initial dose at which the animals could safely be handled (14 ± 4.5 min vs. 12 ± 5.2 min), and the time until the end of the safe handling period (75 ± 12.3 min vs. 85 ± 6.8 min) were similar for both groups. Animals in both groups showed physiological stability during all evaluations, but hypoxemia was observed in one animal in each group. We conclude that both drug combinations are safe and effective at sedating red brocket deer in captivity and suggest that the procedure be performed with oxygen supplementation to reduce the potential for hypoxia.

## Introduction

Red brocket deer (*Mazama americana*) is considered the largest species belonging to the genus *Mazama*, with an average weight between 20 and 40 kg, a height of about 65 cm and a robust appearance [1]. There is a lack of taxonomic data on this species, which is also listed as having “deficient data” with regards its extinction status by the Red List of the International Union for the Conservation of Nature [2]. A strong decline of some populations has been reported [2–4], and there is a need to build knowledge on reproductive barriers [5,6] and cytotaxonomy [7], requiring maintenance in captivity for research and conservation.

Despite being a medium sized animal, red brocket deer cannot be safely handled without chemical immobilization, due to their explosive temperament, enormous strength, and compact body structure [8]. Attempted physical restraint can cause injuries to researchers or to the animal and increase the chances of the animal developing capture myopathy [9,10]. To minimize these risks, it is essential to use immobilizing drugs for chemical restraint, which will enable safe capture and handling procedures in captivity [8,11].

The use of dissociative anesthetics (e.g., ketamine, tiletamine, and telazole) combined with muscle relaxant drugs, are largely used for the chemical immobilization of ungulates, to facilitate safe capture and handling. These drug combinations lead to fast and smooth chemical immobilization induction, and also have a wide safe dose range [12–15]. However, it is noteworthy that dissociative anesthesia makes the animal assume a position of lateral recumbency, coupled with the possibility of hypoxemia and bloating [16–18], prolonged recovery [13,14] and episodes of excitation, and increased muscle tone [19]. Moreover, there are no reversal drugs for dissociative anesthetics. Hence, it is important that chemical immobilization is conducted with drug combinations that promote sedation, muscle relaxation and physiological stability, which can be safely and quickly reversed. Previously, a combination of xylazine and azaperone has been reported to promote sedation and muscle relaxation in pigs without changing other physiological parameters (that would limit the use of this combination) [20]. Moreover, it was found that this combination produced effective sedation in American cervid species without causing other adverse physiological changes [18], and in *Cervus canadensis*, it allowed smooth and safe immobilization, but caused moderate hypoxemia [21].

The aim of this study was to compare the combination of an intramuscular dose of 1 mg/kg azaperone with two different doses of xylazine (0.5 and 1 mg/kg) in captive red brocket deer, in order to obtain the best drug protocol, allowing the performance of routine procedures in captivity without lateral recumbency and cardiorespiratory changes. We hypothesized that the animals would achieve sternal recumbency with cardiorespiratory stability without rough response against the stimuli.

## Materials and methods

Six adult red brocket deer from the Center for Research and Conservation of Cervidae (Núcleo de Pesquisa e Conservação de Cervídeos—NUPECCE) located at UNESP—Campus of Jaboticabal (SP), Brazil (21°14'45.9"S x 48°16'46.3"W) were used in this study. The animals, four males and two females, were aged between 2.5 and 8 years and had a mean weight of 38.4 ± 5 Kg. Five of them were born in captivity and one was born in the wild and hand reared. The animals were kept in individual stalls, with natural light, and water provided *ad libitum*. The feed consisted of dry feed for horses (1 kg) in the morning, and mulberry (*Morus alba*), ramie (*Bohemea livia*), and perennial soybean (*Neonotonia wightii*) leaves in the afternoon. The health of the animals was assessed using prior history, blood counts, serum biochemistry and stool examinations which were performed every 6 months. The study was approved by the Institutional Animal Care Committee of the University of São Paulo (14.1.540.74.4) and by the Environment Ministry (43599–2).

All animals were administered the two proposed immobilizing drug combinations in a randomized order, with > 30 days between treatments: Group AX0.5: 0.5 mg/kg xylazine (Sedomin 10%, König Brasil S.A., São Paulo) combined with 1 mg/kg azaperone (Stresnil 4%, Janssen-Cilag Farmacêutica Ltda., São Paulo); and Group AX1.0: 1 mg/kg xylazine combined with 1 mg/kg azaperone. The deer were fasted 12 hours prior to the administration of each treatment.

On the day of the immobilizing experiment, the animals were guided through corridors to a transport box with small windows for external access. Inside the transport box, the animal was weighed and received the sedative combination intramuscularly in its right or left hind limb (0 minute—basal). The sedative combination was administered by only one person (Duarte JM) through one of the windows in the transport box. Then, the door of the restraint box was opened, and the animals were immediately directed to a 7 m^2^ room, covered with rubberized material on the floor and walls. Through a small window in one of the doors (20 × 20 cm), the animals were observed by a participant who was blind to the treatments (Ferrari BG). By this method, the latency period could be assessed, which was defined as the time between administration of the treatment and the occurrence of ataxia or some sign of sedation behavior. Also, measured in this manner was the time for the animal to assume sternal recumbency, the period for safe handling, and the period from which it was no longer possible to handle the animal safely.

Two observers (Ferrari BG and Silva ANE) performed eight stimuli common in captive deer management, always in the same sequence, every 10 minutes for up to 90 minutes, evaluating the animal’s response to each stimulus. Specifically, the animals were stimulated by (a) touching the ear, (b) clamping the ear using a pair of hemostatic forceps, (c) forced manipulation of the head, (d) pushing the jugular furrow, (e) tail/anal manipulation, (f) insertion of a hypodermic needle into the rump, (g) hoof trimming, and (h) traction of hind limbs.

Physiological parameters were monitored as heart rate by auscultation (HR) (Littmann Classic II S.E. Stethoscope, 3M do Brasil Ltda., São Paulo), mean arterial pressure (MAP) (LifeWindow-LW9xVet, Digicare Biomedical Technology Inc, Florida, USA) by the oscillometric method, respiratory rate (RR) by observation of the ribs, and rectal temperature (OMRON Digital Clinical Thermometer MC-245, Omron Health Care Inc., Illinois, USA). All parameters were evaluated every 10 minutes for 60 minutes or until the safe handling of the animals was not possible.

Blood gas analysis was carried out by measuring the hydrogen potential (pH), arterial partial pressure of carbon dioxide (PaCO_2_), oxygen (PaO_2_), bicarbonate (HCO_3_^-^), base excess (BE), arterial oxygen saturation (SaO_2_), sodium (Na^+^), and potassium (K^+^). To this end, 0.5 mL of blood was collected from the auricular artery at 10, 30, and 60 minutes after the administration of treatments. The samples were analyzed immediately after collection using a blood gas analyzer (I-Stat, Abbott Point of Care Inc., Illinois, USA). The serum lactate concentration was determined from the same samples using a portable lactometer (Accutrend Plus, Roche Diagnóstica Brasil, São Paulo). After completing the evaluations, the animals were transported to their respective stalls, where they remained under observation for two hours or until any risks of trauma or other possible complications were eliminated.

Data were subjected to the Kolmogorov-Smirnov normality test. Data with normal distributions (HR, RR, MAP, rectal temperature, pH, PaCO_2_, PaO_2_, HCO_3_, SaO_2_, Na^+^ e K^+^) were subjected to two-factor analysis of variance (ANOVA) with subsequent Bonferroni test for comparing the groups, and one-way ANOVA with subsequent Tukey’s test for comparing timepoints within each group. A Kruskal-Wallis test, followed by a Dunn test, was used to measure BE and a Mann-Whitney test was used to compare measured lactate, between groups at each time-point and to compare timepoint moments within each group. Periods of latency, sternal recumbency, safe and unsafe handling were subjected to a *t*-test for paired samples. Differences between conditions or timepoints were considered significant at P < 0.05. All parametric data were expressed as mean ± standard deviation and non-parametric data were expressed as median ± interquartile range.

## Results

**Fig 1:**
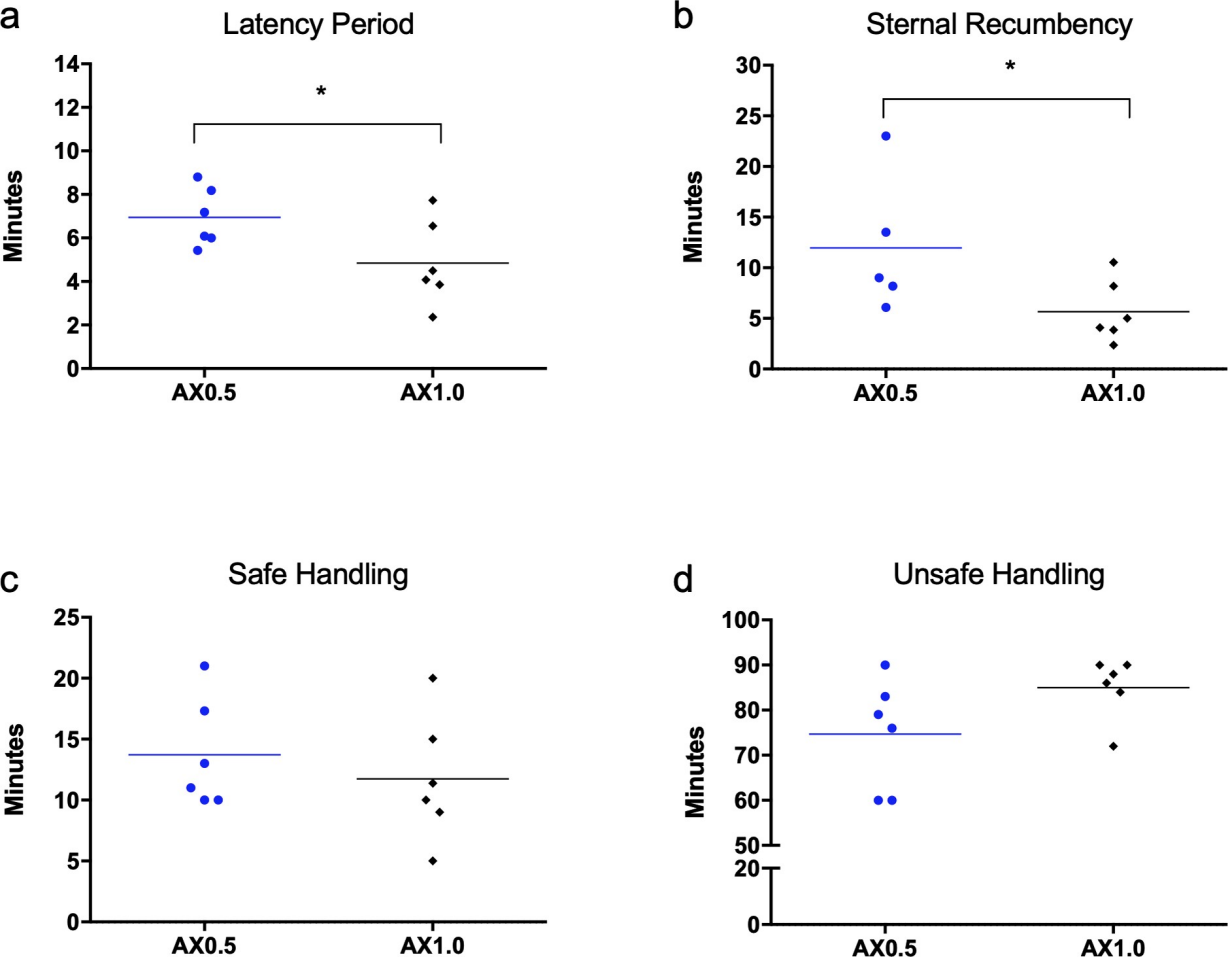
**Latency period (a), for sternal recumbency (b), beginning of ‘safe handling’ (c), and beginning of the moment when handling was no longer safe (d).** Data obtained from red brocket deer given a combination of 1 mg/kg azaperone and 0.5 mg/kg xylazine (AX0.5) or 1 mg/kg xylazine (AX1.0). The dots represent the moment obtained for each animal and the dash represent the mean of the group. *Significant difference between the groups.

All immobilizing drugs were administered safely, using one of the openings in the box, and within 15 seconds of the animal entering the box. The latency period for the animals in the AX0.5 group was longer than that in the AX1.0 group (7 ± 6.6 min and 5 ± 2.0 min, respectively; P = 0.0407). Similarly, the time required for the animals to assume sternal recumbency was more in the AX0.5 group than in the AX1.0 group (12 ± 9.7 min and 6 ± 3.1 min, respectively; P = 0.0235) (Fig 1). The time until safe handling was possible was similar between AX0.5 and AX1.0 (14 ± 4.5 min vs. 12 ± 5.2 min, respectively; P = 0.0680). Also, there was also no difference in the time between the start of the ‘safe handling’ period and the point at which handling became unsafe (75 ± 12.3 min vs. 85 ± 6.8 min, respectively; P = 0.1104).

We also noticed that the temperament of the animals was directly associated with the ease of handling over time in the AX0.5 group. Thus, docile animals allowed contact more easily than the less docile ones. This difference was no longer perceived in group AX1.0, where all animals responded similarly to touch. It is noteworthy that in both groups, the single animal of wild origin had the longest latency time and took the longest to assume sternal recumbency. In addition, one animal in group AX1.0 displayed intense sedation, with interspersed sternal and lateral recumbency, especially when not manipulated by the team. In this case, 0.2 mg/kg yohimbine was administered intramuscularly after 90 minutes of evaluation, reversing the sedative effect of xylazine.

**Fig 2:**
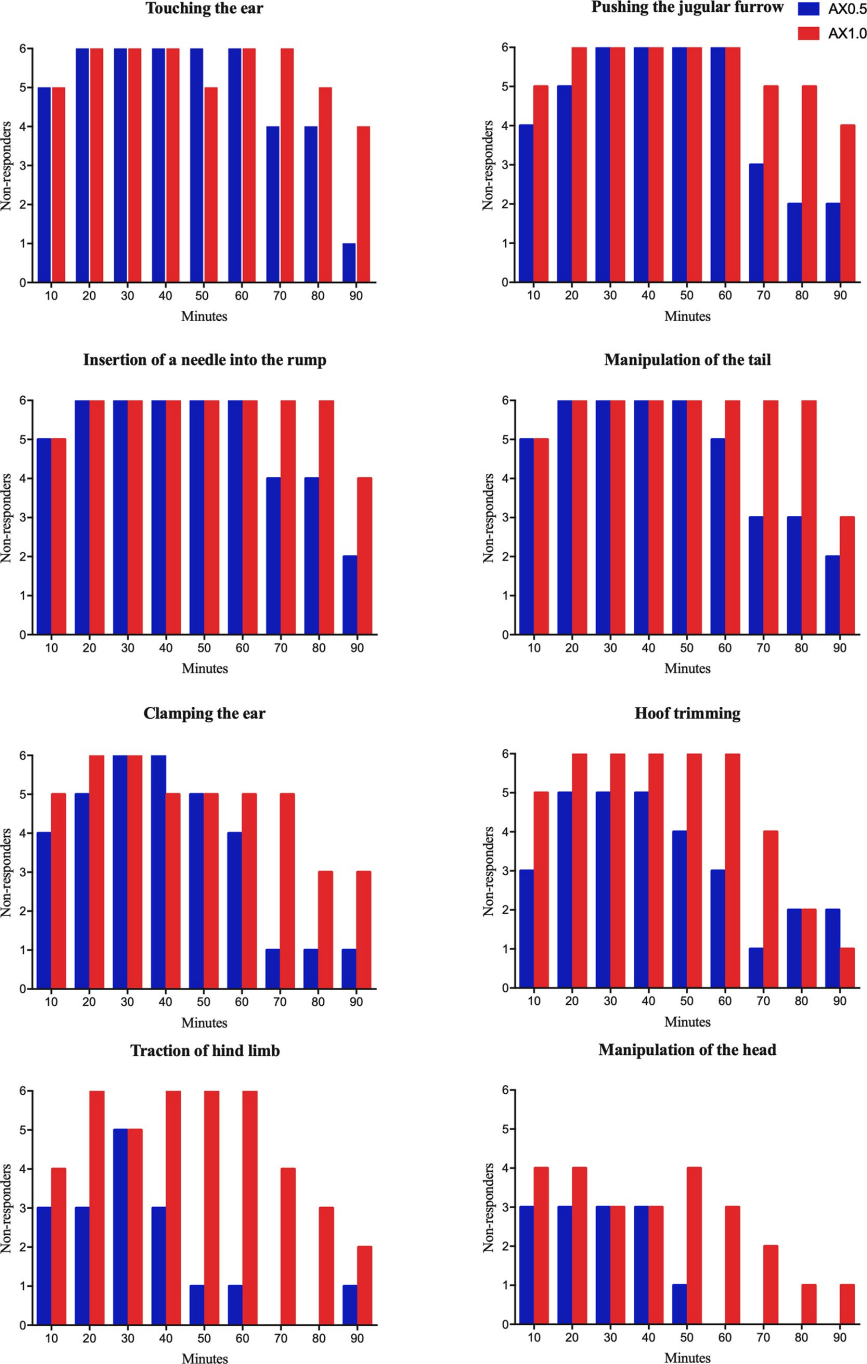
Lack of response of red brocket deer to the stimuli of touching the ear, clamping the ear using a hemostatic forceps, forced manipulation of the head, pushing the jugular furrow, manipulation of the tail, insertion of a hypodermic needle into the rump, hoof trimming, and traction of hind limbs. Data obtained from red brocket deer given a combination of 1 mg/kg azaperone and 0.5 mg/kg xylazine (AX0.5, blue bars) or 1 mg/kg azaperone and 1 mg/kg xylazine (AX1.0, red bars).

Plots of the number of animals that responded to physical stimuli, according to time (Fig 2). Proposed stimuli were determined according to routine management of the animals and the sequence due to the ease of performance. Responses to the eight stimuli can be separated into two distinct blocks. The first, composed of the stimuli of touching the ear, pushing the jugular furrow, tail manipulation, and insertion of a needle into the rump, were easy to perform on the animals between 20 and 60 minutes after treatment. During this period, at least 5 of the 6 animals did not respond to the stimuli, regardless of the treatment received. However, after 60 minutes, we observed increasing responsiveness of the animals in the AX0.5 group, whereas most individuals in the AX1.0 group did not respond to the stimuli until 80 minutes post-treatment.

In the second block of stimuli, considered more intense, we observed that the difference between the treatments resulted in different responses of the animals, especially after 50 minutes after administration (Fig 2). Clamping the ear was possible in at least 5 animals between 20 and 50 minutes, regardless of the group. However, after 50 minutes animals in the AX0.5 treatment displayed a positive response. Both groups had similar results for hoof trimming, between 20 and 40 minutes. The possibility of performing this stimulus was gradually reduced in the AX0.5 group, but was still possible in most animals from AX1.0 to 60 minutes. The limb traction stimulus was only possible in most animals of the AX0.5 group at 30 minutes post-treatment and in animals of the AX1.0 group between 20 and 60 minutes post-treatment. Forced manipulation of the head was the stimulus with the worst results, but even so at least three animals of AX0.5 did not respond up to 40 minutes post-treatment and at least three allowed the stimulus to be performed up to 60 minutes post-treatment.

Neither of the immobilizing protocols promoted marked changes in HR, RR, MAP, and rectal temperature (Table 1). However, it is noteworthy that the animals of group AX1.0 showed bradycardia (values below 60 beats per minute) from 20 to 90 minutes, without needing further treatment. No significant differences in pH values of the blood were observed. Although the blood pH of AX0.5 animals was statistically higher at 60 minutes (7.46 ± 0.03) when compared to 10 minutes (7.38 ± 0.07; P = 0.0447), all values remained within the normal range for this species of deer (Table 1). A similar relationship was observed for the other parameters related to acid-base and electrolyte profiles. However, it should be emphasized that one animal of the AX0.5 group had severe hypoxemia at 10 minutes (PaO_2_ = 57 mmHg) and another of the AX1.0 group had severe hypoxemia at 30 minutes (PaO_2_ = 56 mmHg). Moreover, the AX1.0 animal that experienced hypoxemia was the same animal that assumed lateral recumbency, and had mild bloating which was cured by manually repositioning the animal in the sternal recumbency position.

**Table 1:** Measured physiological parameters of red brocket deer sedated with a combination of 1 mg/kg azaperone and 0.5 mg/kg xylazine (AX0.5) or 1 mg/kg xylazine (AX1.0).

		Minutes					
Parameter	Group	10	20	30	40	50	60
HR (bpm)	AX0.5	71 ± 10.5	69 ± 10.2	66 ± 7.3	62 ± 2.6	62 ± 2.3	67 ± 11.4
	AX1.0	71 ± 19.3	57 ± 5.3	59 ± 12.0	51 ± 11.1	51 ± 11.1	52 ± 6.0
RR (mpm)	AX0.5	59 ± 21.7	52 ± 24.0	52 ± 23.9	46 ± 15.5	45 ± 13.8	45 ± 15.1
	AX1.0	54 ± 21.7	44 ± 9.5	40 ± 13.0	43 ± 19.7	50 ± 16.1	42 ± 7.6
Rectal Temp (°C)	AX0.5	38.9 ± 0.4	38,9 ± 0.4	39.0 ± 0.6	38.9 ± 0.6	38.8 ± 0.6	38.7 ± 0.7
	AX1.0	38.9 ± 0.8	38,8 ± 0.8	39.0 ± 0.8	38.9 ± 0.9	38.8 ± 0.9	38.7 ± 0.8
MAP (mmHg)	AX0.5	72 ± 8.2	80 ± 3.6	83 ± 6.9	76 ± 13.6	69 ± 18.8	61 ± 18.3
	AX1.0	87 ± 16.4	84 ± 16.2	80 ± 6.6	84 ± 7.1	75 ± 15.6	68 ± 15.0
pH	AX0.5	7.38 ± 0.07^a^	-	7.41 ± 0.05 ^a^ ^b^	-	-	7.46 ± 0.03^b^
	AX1.0	7.34 ± 0.15	-	7.37 ± 0.08	-	-	7.46 ± 0.06
PaO_2_ (mmHg)	AX0.5	89 ± 17.4	-	79 ± 12.8	-	-	82 ± 7.8
	AX1.0	83 ± 14.1	-	74 ± 11.6	-	-	89 ± 12.7
PaCO_2_ (mmHg)	AX0.5	29 ± 4.8	-	33 ± 5.0	-	-	35 ± 4.3
	AX1.0	31 ± 5.0	-	35 ± 5.6	-	-	33 ± 4.8
SaO_2_ (%)	AX0.5	95 ± 2.9	-	94 ± 2.6	-	-	95 ± 1.6
	AX1.0	93 ± 3.1	-	92 ± 3.9	-	-	96 ± 2.0
HCO_3_^-^ (mEq/L)	AX0.5	17 ± 4.6^a^	-	21 ± 4,1^ab^	-	-	25 ± 2.8^b^
	AX1.0	17 ± 5.0	-	20 ± 4.7	-	-	23 ± 2.5
BE (mEq/L)	AX0.5	-7		-1.5			2
		[-12.3 –-3.3]^a^	-	[-5.3–0]^ab^	-	-	[-1.0–3.5]^b^
	AX1.0	-5.5		-2.5			0
		[-9.8 –-5.0]^a^	-	[-3.8 –-1,3]^ab^	-	-	[-2.5–1.0]^b^
Na^+^ (mEq/L)	AX0.5	142 ± 1.4	-	141 ± 1.5	-	-	141 ± 2.2
	AX1.0	142 ± 2.3	-	141 ± 3.4	-	-	142 ± 3.3
K+ (mEq/L)	AX0.5	3.6 ± 0.2^a^	-	3.8 ± 0.2^ab^	-	-	4.2 ± 0.6^b^
	AX1.0	3.7 ± 0.4.	-	3.9 ± 0.1	-	-	4.2 ± 0.6
Lactate (mmol/L)	AX0.5	-	-	3.7 [1.3–6.2]	-	-	1.6 [1.0–2.3]
	AX1.0	. -	-	2.6 [2.0–3.4]	-	-	1.3 [0.9–1.7]

Different letters represent statistical difference. Results expressed as mean ± standard deviation, except for BE, which was represented as median ± interquartile range.

Though stable in both treatment groups, serum lactate was numerically higher at 30 minutes when compared with values at 60 minutes. Interesting, the wild born animal had higher had higher values compared to the others after the administration of treatment AX1.0, as 13.5 mmol/L at 30 min and 8.8 mmol/L at 60 minutes.

## Discussion

Red brocket deer are difficult to manage [8] and the standardization of a chemical restraint protocol that allows simple procedures to be carried out and avoids lateral recumbency is highly important. Our aim was therefore to obtain adequate chemical restraint, enabling safe handling of the animals with a view to facilitate the main activities performed in a classic system of maintenance for medium sized cervids in captivity (www.youtube.com/watch?v=Ipzszmu3saI&feature=youtu.be).

The latency period and time before the occurrence of sternal recumbency were significantly longer in the animals of the AX0.5 group than in the animals of the AX1.0 group. This was expected due to the difference in the dose of xylazine between both groups. It is important to consider the difference between wild and captive animals, namely that captive animals show less explosive temperaments and consequently are less subject to stress than wild animals [22–24]. The difference between the latency period of both treatment groups may be greater in animals that are poorly adapted to captivity situations.

Despite the difference in the latency period and sternal recumbency, we did not observe any significant difference in the time elapsed between the administration of drugs and the possibility of safely handling the animals. Most animals allowed safe handling within 15 minutes after administration of the drugs, and all within 20 minutes, regardless of the treatment administered. Longer times have previously been reported in white-tailed deer (*Odocoileus virginianus*) [23], due to the lower dose of azaperone (0.37 mg/kg), interspecies difference, and because they were wild animals.

Although the period for safe handling of the animals occurred only after 20 minutes, we consider this a reasonable waiting time when it comes to handling wild cervids, even in captivity. Furthermore, we found that most management procedures could be performed for 40 minutes (20 to 60 minutes after administration) in both groups: a relatively comfortable time period. The fact that to our knowledge no studies exist that evaluate this period of sedation in cervids with the proposed combination makes it difficult to compare the results obtained, since those studies that are available studies used reversible protocols in cervids [22,23,25], thus having a different objective from ours.

Non-response to touch or clamping of the ear, pushing the jugular furrow, tail manipulation, or insertion of a needle into the muscles of the hind limb was an important finding of our study. We show clearly that several routine management procedures can be performed in these animals using of any of the chemical immobilization protocols. These include the collection of blood, mucosal cells (ocular, oral, vaginal, and preputial) or hair, as well as the ability to perform detailed clinical examination, markings with earrings or microchips, biometry, the administration of intravenous drugs and more.

We conclude that more intense management procedures, such as trimming and cutting of horns, should be performed after the administration of 1 mg/kg azaperone combined with 1 mg/kg xylazine, IM, which allowed the manipulation of hind limbs, without animal reaction, for more than 40 minutes (20 to 60 minutes after administration) and in most cases (4 out 6 animals) allowed safe handling of the head for at least 20 minutes. We emphasize that hoof trimming and horn cutting are extremely important management behaviors in captivity, mainly due to the accentuated growth of the hooves in captive systems and the aggressiveness of the males towards humans and female red brocket deer.

The animals maintained relative cardiorespiratory stability during all evaluations. However, the animals of group AX1.0 showed bradycardia (HR < 60 bpm) from the 20 minute timepoint onwards. This effect was probably a reflex to initial hypertension due to increased systemic vascular resistance previously reported after the administration of alpha_2_-adrenergic agonists such as xylazine [26–28]. Another possibility is that the decrease in the sympathetic tonus promoted by xylazine inhibited the release of noradrenaline [26]. Statistical differences in mean arterial pressure were not observed. However, two animals from each group had values below 60 mmHg, which may have been caused by the possible decrease in sympathetic tonus caused by xylazine [26], and because azaperone acts on the vascular smooth muscle, causing peripheral vasodilatation [29].

Blood gases and electrolytes remained stable and in the acceptable range for cervids in situations of resting and chemical restraint [21,30]. Intercurrences such as hypoventilation and hypercapnia were not observed in our study, although they are common in chemical restraint of cervids [18,28]. Only one animal of the AX1.0 group had mild bloating at 40 minutes, but without considerable increase in PaCO_2_, which was relieved by repositioning the animal to the sternal recumbency position [23,31].

Cardiorespiratory monitoring in wild animals undergoing chemical restraint is often inadequate, being based only on HR, RR, and pulse oximetry. Although we did not observe changes in these parameters in either group, most animals had moderate hypoxemia (PaO_2_ between 60 and 80 mmHg), at least at some points during the evaluations. It is noteworthy that one animal had severe hypoxemia (PaO_2_ < 60 mmHg) in both groups, at 10 minutes in the AX0.5 group and at 30 minutes in the AX1.0 group, which were not sufficient to cause statistical differences in PaO_2_ values, probably due to the small sample size.

Hypoxemia is generally not diagnosed in wild animals undergoing chemical restraint, because blood gases are rarely evaluated in field conditions [30]. One strategy that could decrease the possibility of hypoxemia would be oxygen supplementation [22,32]. However, similar to other studies [13,21,23], we did not use any source of oxygen supplementation in the animals of this study. Although hypoxemia was observed in some animals, the values were normalized in a period considered acceptable, as observed in *Cervus canadensis* [21]. However, we strongly recommend the use of oxygen supplementation during the immobilization procedure, as a precaution.

## Conclusion

The two protocols used were effective for chemical restraint of the red brocket deer. We recommend that a high or low dose of xylazine, combined with azaperone, be chosen based on the kind of management to be performed and the nature of the animal kept in captivity. Moreover, although restraint did not cause considerable physiological changes, we strongly recommend oxygen supplementation during the restraint process.
